# Characteristic of patients with disorders of sex development (DSD) in Cipto Mangunkusumo Hospital, Jakarta

**DOI:** 10.1186/1687-9856-2013-S1-P193

**Published:** 2013-10-03

**Authors:** Agustini Utari, Aman B Pulungan, Bambang Tridjaja

**Affiliations:** 1 Division of Endocrinology, Department of Pediatrics, Faculty of Medicine, Diponegoro University/ Dr. Kariadi Hospital, Semarang, Indonesia; 2 Division of Endocrinology, Department of Pediatrics, Faculty of Medicine, University of Indonesia/ Cipto Mangunkusumo Hospital, Jakarta, Indonesia

## 

Disorder of Sex Development (DSD) is a congenital condition in which development of chromosomal, gonadal or anatomical sex is atypical. They are complex condition related to social stigma, cultural and beliefs in Indonesian population. In Indonesia, there is only a few study regarding characteristic of DSD patients. The aims of our study was to evaluate the characteristic of patients with DSD in Cipto Mangunkusumo Hospital, Jakarta, Indonesia.

We retrospectively reviewed the medical records of patients followed up over the period from 2010 to 2012 in Endocrinology Pediatric Clinic, Cipto Mangunkusumo Hospital, Jakarta, Indonesia. We found 74 patients with DSD. In this paper we excluded 29 patients confirmed as Congenital Adrenal Hyperplasia and 3 patients with Turner syndrome.

Of 42 patients, 23 (54.8%) patients had 46,XY DSD; 11 (26.2%) patients had sex chromosome DSD; 3 (7.1%) patients had 46,XY DSD, and 5 patients have not done chromosomal analysis. The age when first diagnosed was ranging from newborn to 14 years old. Most cases referred by pediatrician (33.3%) and urologist (23.8%) with frequent complaint were ambiguous genitalia (52.4%) and severe hypospadia (14.3%). The most frequent of phenotype was hypospadia (66.7%). Twenty five (59.5%) of patients have been raised as boy prior to diagnosis. Twelve patients were raised as girls have 46, XY karyotype and 2 patients raised as boys have 46, XX karyotype. Definite diagnosis of 23 patients with 46, XY DSD was found only in 4 (17.4%) patients. The problem regarding lack of definitife diagnosis was related with the high cost of laboratory examination and socioeconomic problem in Indonesia.

Our study suggest that the most common cause of DSD in our clinic was 46, XY DSD. Karyotype and clinical phenotype may help in establishing the diagnosis.

